# Runx3 Mediates Resistance to Intracellular Bacterial Infection by Promoting IL12 Signaling in Group 1 ILC and NCR+ILC3

**DOI:** 10.3389/fimmu.2018.02101

**Published:** 2018-09-12

**Authors:** Shengxia Yin, Jingjing Yu, Bian Hu, Chenyu Lu, Xia Liu, Xianzhi Gao, Wei Li, Lina Zhou, Jianli Wang, Di Wang, Linrong Lu, Lie Wang

**Affiliations:** ^1^Institute of Immunology, and Bone Marrow Transplantation Center of the First Affiliated Hospital, Zhejiang University School of Medicine, Hangzhou, China; ^2^Institute of Hematology, Zhejiang University & Zhejiang Engineering Laboratory for Stem Cell and Immunotherapy, Hangzhou, China; ^3^School of Life Science and Technology, ShanghaiTech University, Shanghai, China; ^4^State Key Laboratory for Diagnosis and Treatment of Infectious Diseases, Collaborative Innovation Center for Diagnosis and Treatment of Infectious Diseases, The First Affiliated Hospital, College of Medicine, Zhejiang University, Hangzhou, China; ^5^Department of Laboratory Medicine, The First Affiliated Hospital, College of Medicine, Zhejiang University, Hangzhou, China; ^6^Laboraty Animal Center, Zhejiang University, Hangzhou, China; ^7^Institute of Immunology, Zhejiang University School of Medicine, Hangzhou, China

**Keywords:** ILCs, RUNX3, IL12 signaling, Intracellular bacterial infections, mouse models

## Abstract

Innate lymphoid cells (ILCs) are the most recently identified family of the innate immune system and are hypothesized to modulate immune functions prior to the generation of adaptive immune responses. Subsets of ILCs reside in the mucosa and regulate immune responses to external pathogens; however, their role and the mechanism by which they protect against intracellular bacterial infection is not completely understood. In this report, using *S. typhimurium* and *L. monocytogenes*, we found that the levels of group 1 ILCs and NCR^+^ ILC3s were increased upon infection and that these increases were associated with Runt-related transcription factor 3 (Runx3) expression. Runx3 ^fl/fl^ PLZF-cre mice were much more sensitive to infection with the intracellular bacterial pathogens *S. typhimurium* and *L. monocytogenes* partially due to abnormal Group 1 ILC and NCR^+^ILC3 function. We also found that Runx3 directly binds to the *Il12R*β*2* promoter and intron 8 to accelerate the expression of Il12Rβ2 and modulates IFNγ secretion triggered by the IL12/ STAT4 axis. Therefore, we demonstrate that Runx3 influences group 1 ILC- and NCR+ILC3-mediated immune protection against intracellular bacterial infections of both the gut and liver.

## Introduction

Innate lymphoid cells (ILCs) are newly discovered constituents of the innate immune system, which develop from the lymphoid lineage. Based on their expression of key transcription factors and production of cytokines, ILCs are classified into three distinct groups. Group 1 ILCs are T-bet-expressing cells that secrete interferon (IFN)-γ, similar to type 1 T helper (Th1) cells. ILC2s are characterized by their expression of GATA-3 and capacity to secrete type 2 T helper (Th2) cell cytokines, including interleukin-4 (IL-4), IL-5, IL-9, and IL-13, whereas ILC3s express retinoid-related orphan receptor γt (RORγt) and produce IL-17 and/or IL22, similar to Th17 and Th22 cells (1).

One important characteristic of ILCs, which underscores their role in the immune system, is that they are located in barrier tissues, allowing them to rapidly react to bacteria, helminths and viral infections. Group 1 ILCs are divided into two groups: natural killer (NK) cells and ILC1s, mainly depending on the expression of the transcription factor Eomesodermin (Eomes) (2). They typically accumulate in the intestine (3), liver (4) and salivary glands (5) and secret functional cytokines in response to upstream signals, such as IL-12, IL-15, or IL-18 (6, 7). They were reported to protect mice against the protozoan intracellular parasite *Toxoplasma gondii* by producing high levels of IFNγ and tumor necrosis factor alpha (TNFα) (7), and they were linked to IFNγ -dependent recovery from acute infection with the opportunistic enteric pathogen *Clostridium difficile* in mice (8). Moreover, ILC1-derived IFN-γ limits early mouse cytomegalovirus (MCMV) replication in infected primary tissues (9). ILC3s are divided into two groups, NCR^−^ILC3s and NCR^+^ILC3s, depending on the expression of natural cytotoxicity triggering receptors (NCRs) (10). They are mainly distributed in the gut to maintain homeostasis (11) and combat infection by secreting IL17, IL22, and IFNγ. It was reported that ILC3 driven IL-22 production has crucial role in the early phase of the host defense against *C. rodentium*. The infection of IL-22 knockout mice results in increased intestinal epithelial damage, systemic bacterial burden and mortality (12–14). Moreover, in *Helicobacter hepaticus* (Hh)-driven colitis, ILC3s accumulate in the inflamed colon and contribute to colitis through IL-23–driven IL-17 and IFN-γ production (15).

Several transcription factors were demonstrated to affect the function of multiple ILC subsets. A number of groups described defects in multiple ILC subsets in nuclear factor interleukin 3 regulated (NFIL3)-deficient mice, including loss of IFNγ^+^ intestinal ILC1s and reduced numbers of ILC3s (16), leading to impaired mucosal defenses. GATA3 binds to the promoter of *IL22* to promote the secretion of IL22 in ILC3s (17). T-bet is the master transcriptional regulator of ILC1 cells and regulates production of the ILC1 effector cytokines IFNγ and TNFα. Furthermore, T-bet modulates the secretion of IL22 in NCR^+^ILC3s. RORγt acts as a master regulator for all ILC3 cells, including CCR6^+^ LTi/LTi-like cells and NKp46^+^ ILC3s. RORγt is also responsible for the production of ILC3 effector cytokines, such as IL-22, IL-17a, and IL-17f (18). Ahr is a ligand-dependent transcriptional factor, which senses environmental and endogenous compounds generated by commensal, dietary, or cellular metabolism. It was reported that Ahr is essential for the maintenance and production of IL-22 by ILC3 (19). Additionally, Ahr-deficient mice succumb to *Citrobacter rodentium* infection, and ectopic expression of IL-22 protects animals from early mortality (14).

Runx3 is a transcription factor belonging to the Runx family and is characterized by expressing an evolutionarily conserved DNA-binding Runt domain (7). The role of Runx3 in controlling thymocyte differentiation and the CD4/CD8 lineage decision is well established (20–22). Additionally, Runx3 was reported to have a role in cytotoxicity. In particular, Runx3 is highly expressed and interacts with T-box transcription factor (T-bet) to form a transcription complex that promotes the expression of IFNγ in Th1 differentiation. Some reports support the hypothesis that Runx3 alone is sufficient to induce strong IFNγ secretion in both Th1 cells and CTL cells (23). In addition, it was reported that Runx3 was expressed in both intestinal group1 ILC and ILC3, and Runx3 also plays an essential role in the development of both ILC1 and ILC3 (24). Furthermore, in the intestine of Runx3^f/f^ NKp46 cre mice, the number of both ILC1 and NCR^+^ILC3 cells was significantly decreased. The above results indicate that expression of Runx3 in group1 ILC and ILC3 may have a role in protection against intracellular bacteria.

In this report, using *S. typhimurium* and *L. monocytogenes* infection models, we found that after infection, the number of cells and expression of Runx3 for group1 ILC and NCR^+^ILC3 were increased in tissue. To address the function of Runx3 function in group 1 ILC and NCR+ILC3 against intracellular bacterial infection, we generated Runx3-deficient mice by crossing mice with loxP-flanked Runx3 alleles (Runx3^fl/fl^) with PLZF-cre mice. We found that these mice were more sensitive to infection induced by the intracellular bacteria *S. typhimurium* and *L. monocytogenes*, to some extent, due to the abnormal function of group 1 ILCs and NCR+ILC3s. To further elucidate the underlying mechanism, we found that the expression level of IL12Rβ2 was decreased upon deletion of Runx3 in ILC1s and NCR+ILC3. Moreover, Runx3 directly binds to the *IL12R*β*2* promoter and intron 8 to accelerate the expression of IL12R and modulate the IL12/ STAT4 axis to trigger IFNγ secretion. Therefore, our data reveal a previously unknown protective function of Runx3 in orchestrating innate immunity against intercellular bacterial infection.

## Materials and methods

### Mice

The Plzf-Cre-GFP strain was generated as follows: the sequence encoding a Cre-GFP-FRT-Neo-FRT cassette was inserted immediately after the start codon of *Zbtb16* by homologous recombination. The linearized construct was transfected into C57BL/6J embryonic stem cells and neomycin-resistant clones were confirmed by PCR and Southern blotting. Clones that had undergone homologous recombination were injected into albino C57BL/6J blastocysts and the resulting chimeric mice were crossed with Gt(ROSA)26Sor^tm1(FLP1)Dym^ (also known as ROSA26 FLPe knockin) mice to excise the neomycin resistance cassette. The following PCR primers were used to identify WT (311 base pairs) and knock-in (581 base pairs) alleles: 5′-AGTTGTTGTCACTTGCTCACCC-3′, 5′-GTGAACGAACCTGGTCGAAAT-3′, and 5′-CTTGCTGGTGCAGGCTAGCA-3′. The Plzf-Cre-GFP mice were then backcrossed onto the C57BL/6 background for five generations. Runx3^F/F^ mice were purchased from Jackson Laboratories (Bar Harbor, ME, USA). NOD-Prkdc^em26Cd52^Il2rg^em26Cd22^/Nju (NCG, T001475) mice were purchased from Nanjing Biomedical Research Institute of Nanjing University.

All procedures involving animals were approved by the laboratory animal center of Zhejiang University.

### *S. typhimurium* and *L. monocytogenes* infection

To induce *S. typhimurium* intestinal infection, mice were fasted for 4 h and received 20 mg streptomycin per mouse by oral gavage, while control mice were administered with an equivalent volume of vehicle containing pyrogen-free PBS, before infection. 20 h later, 10^9^ *S. typhimurium* (SL1344, SB300) or equivalent volume of pyrogen-free PBS was taken to infect mice via oral gavage. 4 days later, the infected mice were sacrificed for further study. The small intestines were used to isolate intestinal lymphocyte cells and the colon was fixed for 24 h in 4% methanol for H&E staining.

To induce *L. monocytogenes* liver infection, mice were administrated with 10^5^ CFU of *L. monocytogenes* (10403s) or equivalent volume of pyrogen-free PBS by tail vain injection. 48 h after the infection, mice were sacrificed, and the livers were used for following experiments.

### Isolation of intestinal lymphoid cells

Mouse small intestines were cut open longitudinally after removing Peyer's patches, and washed with DMEM, and these were then cut into pieces about 5 mm in length. The intestine pieces were incubated in pre-warmed DMEM containing 3% FBS, 0.2%Hanks, 5 mM EDTA, and 0.145 mg/ml DTT for 10 min with constant agitation by droppers. The dissociated cells were collected as IELs. Then the small intestine was incubated in a solution of 3% DMEM, 0.2% FBS, 0.025% Hanks, 50 mg/ml DNase and 75 mg/ml collagenase II for 5 min and the dissociated cells were collected as lamina propria lymphocytes. Finally, the solution containing digested tissue was passed through a 100 μm cell strainer and LPLs were isolated by an 80/40% Percoll (GE Healthcare) gradient.

### T cell activation

Antigen-presenting cells (APCs) were obtained from C57BL/6 splenocytes by a FACSAria III sorter (BD) and irradiated at 2500 rad. Sorted CD44^lo^ CD4^+^CD8^−^ or CD44^lo^ CD4^−^CD8^+^LN T cells (0.5 × 10^6^) were mixed with 2 × 10^6^ irradiated APCs in complete culture medium (RPMI 1640 supplemented with 10% FCS) and activated with anti-CD3 (145-2C, 1 μg/ml) and anti-CD28 (E18, 3 μg/ml) in the presence of either 50 U/ml IL-2 (“CD8 conditions”) or 50 U/ml IL-2, 10 ng/ml IL-12, 10 μg/ml anti-IL-4 (“Th1 conditions” All cytokines were from Preprotech.

### Flow cytometry and antibodies

The following eBiosciences antibodies were used in our experiments: CD4 (RM4-5, 1 mg/ml), CD8a (53-6.7, 2.5 mg/ml), TCRb (H57-597, 1 mg/ml), NK1.1 (PK136, 1 mg/ml), CD24 (M1/69, 1 mg/ml), TCRgd (GL3, 1 mg/ml), CD11b (M1/70, 1 mg/ml), CD11c (N418, 1 mg/ml), CD122 (TM-b1, 1 mg/ml), Gr-1 (RB6-8C5, 1 mg/ml), Ter119 (TER-119, mg/ml), CD19 (eBio1D3, 1 mg/ml), RORgt (B2D, 2.5 mg/ml), Eomes(Dan11mag, 1 mg/ml), Runx3(R3-5G4, 1 mg/ml), PLZF(B263557, 1 mg/ml) TNFα(MP6-XT22, 1 mg/ml), IFNγ(XMG-1.2, 1 mg/ml), p-STAT4(4LURPIE, 1 mg/ml), IL12Rβ2(305719, 1 mg/ml), IL18Rα(70625, 1 mg/ml), IL15R(eBioJM7A4, 1 mg/ml), NKp46(9E2, 1 mg/ml) and T-bet (4B10, 1 mg/ml). The PBS57-loaded and unloaded (control) mouse PE-conjugated CD1d tetramer and isotype control were obtained from the NIH Tetramer Core Facility, USA. FITC annexin V (1:20 dilution) was from BioLegend. Flow cytometry was performed with Fortessa and FACSAria II machines (BD Biosciences). Data were analyzed with FlowJo software (Tree Star, Inc.). Intracellular staining was processed using IC fixation buffer (eBiosciences), A Foxp3/transcription factor staining buffer set (eBiosciences) was used for RORgt and cytokine staining. Cells were sorted by a FACSAria II flow cytometer.

Group 1 ILC cells from sLPL were sorted as CD3-CD19-NK1.1+NKp46+ cells and analyzed as CD3^−^CD19^−^CD45^+^NK1.1^+^NKp46^+^Eomes^+^ (NK cell) and CD3^−^CD19^−^CD45^+^NK1.1^+^NKp46^+^Eomes^−^ (ILC1 cell). ILC2 from sLPL were analyzed as CD3^−^CD19^−^CD45^+^CD127^+^KLRG1^+^ and ILC3s from sLPL were stained as CD3^−^CD19^−^CD45^+^CD127^+^ RORgt^+^. Group 1 ILCs from liver were analyzed as CD3^−^CD19^−^CD45^+^NK1.1^+^NKp46^+^Eomes^+^ (NK cell) and CD3^−^CD19^−^CD45^+^NK1.1^+^NKp46^+^Eomes^−^ (ILC1 cell). CLPs from bone marrow were analyzed as Lin^−^Sca1^int^c-kit^int^ CD127^+^Flt3^+^, CHILP as Lin^−^CD127^+^a4b7^+^CD25^−^Flt3^−^ and iILC2 as Lin^−^CD127^+^a4b7^+^CD25^+^Sca1^+^. Dead cells were excluded by 4,6-diamidino-2-phenylindole staining.

### Adopt-transplantation of ILC to NCG mice

6000 ILCs (CD3^−^CD19^−^CD11c^−^CD90^+^NK1.1^+^ NKp46^+^) were purified from the sLPL of naive cKO and control mice by a FACSAria III sorter (BD). Sorted ILCs were incubated in 1640 with 10% FBS, 40 ng/ml IL-12 and 40 ng/ml IL-18 at 37°C for 1 h to support cell viability and optimal IFNγ production before i.v. transferring into recipient NCG mice on days −1, 0, 1 and 5 day after S.typhimurium infection orally. 9 days after the infection, mice were sacrificed for further analysis.

### Generation of cells constitutively express 3xflag-Runx3

EL4 (ATCC® TIB-39™) and retroviral packaging cell lines Plat-E were cultured in DMEM with 10% FBS and 1% antibiotics. All cultures were grown at 37°C in a humidified 5% CO_2_-air atmosphere.

Retrovirus preparation was performed in Plat-E cells. Plat-E cells were transfected with pMX-IRES-GFP containing 3xflag tagged Runx3 gene, the medium was replaced with fresh medium after 10 h, and retrovirus supernatant was collected after additional 72 h.

EL4 cells were spun in 1 ml of virus supernatant with 8 μg/ml polybrene (Sigma) at 2,500 g for 2 h at 32°C. The retroviral transduction was repeated 24 h later. Five days later, GFP positive cells were sorted by flow cytometry and constitutively expressing flag labeled Runx3 were cultured for further analysis.

### Luciferase reporter assay

The promoter and intron8 of IL-12Rβ2 and their mutants were produced by PCR-based amplification and subcloned into the pGL3-Enhancer Vector to form a luciferase reporter plasmid.

Human embryonic kidney (HEK293) cells were co-transfected with 100 ng of the luciferase reporter plasmid, 10 ng of a thymidine kinase promoter-Renilla luciferase reporter plasmid, plus the pCDNA3-Runx3 or control vector. After 48 h, luciferase activities were determined by the Dual-Luciferase Reporter Assay System (Promega, Cat. No. E10910) according to the manufacturer's instructions.

The primers were as followed:

*Il12rb2*-promotor-WT: ACCAAGGATTTCCACAGCTCA
TAAGTTATTATGCAAGAACTACA
*Il12rb2*-intron 8-WT: CTCGAGAAAGCTACCTGCCAATC
AGAA
AAGCTTCACTTCATCTACCTTGT
ATTAG

We synthesized the whole gene fragment of *Il12rb2*-promotor-MUT and *Il12rb2*-intron 8-MUT by mutating the Runx3 binding site CCACA into TTTCA.

### Plasmid constructs

A recombinant vector encoding mouse Runx3 was constructed by PCR-based amplification and subcloned into the pcDNA3 eukaryotic expression vector. Recombinant vectors encoding mouse 3xflag-Runx3 and IL12Rβ2 were constructed by PCR-based amplification and subcloned into pMX-IRES-GFP eukaryotic expression vector.

### CHIP assay

10^5^ EL4 cells constitutively expressing 3xflag-Runx3 or control were fixed with 1% formaldehyde. The crosslinked chromatin was sonicated in a 4°C water bath using Bioruptor UCD-200 sonicator to obtain DNA fragments sized between 100 and 200 bp. Flag antibody linked beads were used to pull down DNA binding Runx3 at 4°C over night. The protein-DNA binding fragments were digested by proteinase for 4 h before applying the DNA fragments into an RT-PCR assay.

The ChIP qPCR primers were listed as follows:

*Il12rb2*-promotor: ACCAAGGATTTCCACAGCTC
ATAAGTTATTATGCAAGAACTACA
*Il12rb2*-intron 8: CTTGCTTTTCCAGTTTGATCTT
AAGAGTGGTAAGGGGTGTAAA
*Ifng*: GCTTTCAGAGAATCCCACAAGAAT
GCTATGGTTTTGTGGCATGTTAGA
Actin: GTGTTAGGGAGGCTTGATC
GAACGGCAGCCACTTGTG

### Cytokine stimulation

The sorted T cells were seeded in 96-well plate and stimulated with PMA (25 ng/ml) to analyse the introcular INFγ, stimulated with PMA (25 ng/ml) and ionomycin (500 ng/ml) to analyse the introcular TNFα. The sorted LPLs were seeded in 96-well plate and stimulated with PMA (25 ng/ml) and ionomycin (500 ng/ml) or the indicated cytokines. Cells were cultured at 37°C for 4 h in RPMI 1640 medium supplemented with 10% FBS and used in the following experiment after PBS wash.

### ELISA assay

For *in vitro* IFNγ detection, group 1 ILC cells were sorted from sLPL and seeded 2,000 per well. Cells were stimulated for 72 h with recommended cytokine concentrations of 40 ng per well in 96-well plates with DMEM (C0006; Gibco, Carlsbad, CA, USA) and 10% FBS (Gibco, Carlsbad, CA, USA) at 37°C under 5% CO_2_. The supernatants were then collected and measured by ELISA using Ready-Set-Gokits (eBioscience, San Diego, CA, USA) for cytokine determination. All assays were performed in triplicate.

### IL12Rβ2 rescue

Expression of ectopic proteins in group1 ILCs was performed by using RetroNectin (Takara) kit. In brief, RetroNectin (Takara) coating and washing were carried out according to the manufacturer's instruction. The retrovirus was added to wells coated with RetroNectin, followed by 4 h incubation at room temperature and removal of the retrovirus. After 24 h co-culture, sorted group1 ILCs from both cKO mice and their wild type compartments were directly placed on plates coated with RetroNectin and retrovirus. Trypsinized OP9-DL1 cells were added to the group1 ILCs cells. Forty-eight hour later, cells were stimulated with IL12 and IL18 and the secretion level of IFNγ was analyzed by ELISA.

### Histological analysis

To determine the degree of injury, the livers, small intestine and colon were fixed in 4% paraformaldehyde and embedded in paraffin. Sections (4-μm) were cut and stained with hematoxylin and eosin.

### Statistical analyses

Data were analyzed by two-tailed Student's *t*-test with Graphpad Prism 5. *P*-values of < 0.05 were considered statistically significant. No randomization was used in animal studies. No deliberate attempt was made to study only selected mice except based on genotype.

## Results

### Group 1 ILC and NCR^+^ILC3 accumulate in organs infected with intracellular bacteria and upregulate the expression of Runx3

The murine models of systemic *L.monocytogenes* and *S. typhimurium* infections are excellent experimental systems to study immune responses against intracellular pathogens. *L. monocytogenes* and *S. typhimurium* are facultative, intracellular organisms. *L.monocytogenes* can invade the liver and spleen of the host (25), whereas *S. typhimurium* mainly infects host through intestinal epithelial cells and causes diarrhea (26). Via tail vain injection of *L. monocytogenes* or oral infection with *S. typhimurium*, we tested the response of liver group 1 ILC, intestinal group 1 ILC and NCR^+^ILC3 cells.

**Figure 1:**
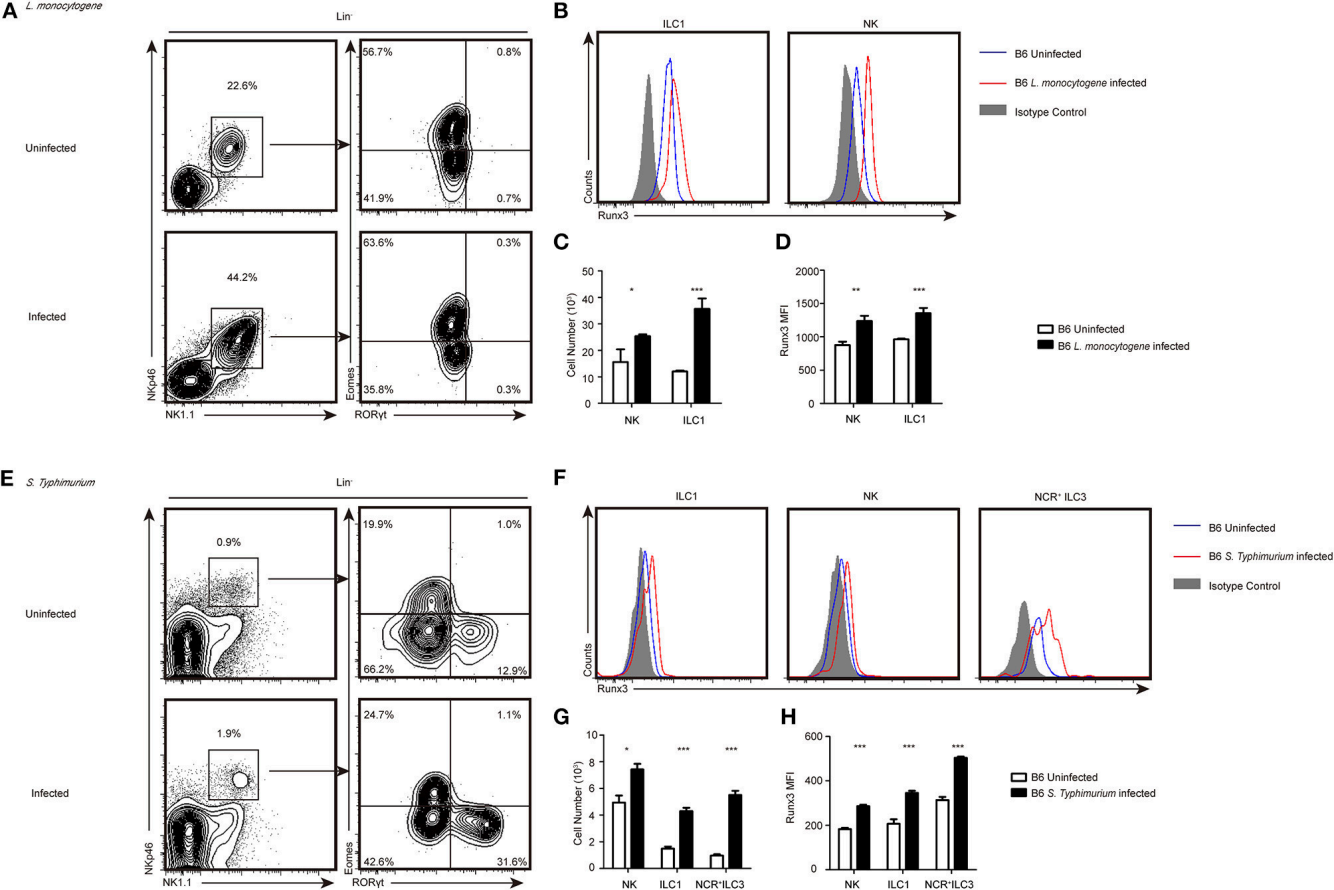
Group 1 ILCs and NCR^+^ILC3s accumulated intracellularly in bacteria infected organs and upregulated the expression of Runx3. **(A–D)** B6 mice were infected with *L. monocytogenes* through tail vein injection as the infected group (*n* = 3) or injected with sterile PBS as the uninfected group (*n* = 3). **(A)** Flow cytometry assay of the percentage of ILC1s and NKs from the livers of both groups. ILC1s were stained Lin^−^NK1.1^+^NKp46^+^RORγt^−^Eomes^−^ and NKs were Lin^−^NK1.1^+^NKp46^+^ RORγt^−^Eomes^+^. **(B)** The expression of Runx3 was analyzed by flow cytometry in ILC1s and NKs from the liver. The isotype controls are the shaded curves, uninfected groups are blue curves and infected groups are red curves. **(C)** Absolute number of total ILC1s or NKs from livers before and after infection. **(D)** Flow cytometry analysing the expression of Runx3 in the indicated cell types. **(E–H)** B6 mice were infected with *S. typhimurium* by gavage administration as the infected group (*n* = 3) or lavaged with sterile PBS as the uninfected group (*n* = 3). The isotype controls are shaded curves, uninfected groups are blue curves and infected groups are red curves. **(E)** Flow cytometry assay of the percentage of ILC1s, NKs and NCR^+^ILC3s in both groups. NCR^+^ILC3s were stained Lin^−^NK1.1^+^NKp46^+^RORγt^+^Eomes^−^. **(F)** The expression of Runx3 was analyzed by flow cytometry in ILC1s, NKs and NCR^+^ILC3s from the intestine. **(G)** Absolute number of total ILC1s, NKs or NCR^+^ILC3s from the intestines before and after infection. **(H)** The expression of Runx3 in ILC1s, NKs and NCR^+^ILC3s from the intestine (mean ± SD of three samples in **(C,D,G,H)**; ^*^P < 0.05, ^**^P < 0.01 and ^***^P < 0.001 by Student's *t*-test). Data are from one experiment representative of three independent experiments with similar results in **(A,E)**.

At 48 h after tail vein injection of *L. monocytogenes*, both the relative percentage and total cell number of group 1 ILCs increased in the liver (Figures 1A,C). We further assessed the protein level of Runx3 in group 1 ILC from the liver and showed that Runx3 was significantly increased in hepatic ILC1s and NK cells (Figures 1B,D). Consistent with the *L. monocytogenes* model, we found a significant increase in both the percentage and number of ILC1s and NCR^+^ILC3 cells in the lamina propria of the murine small intestine 3 days after oral infection with *S. typhimurium* (Figures 1E,G). Elevated Runx3 levels were also observed in ILC1s and NCR^+^ILC3 from the lamina propria of the murine small intestine after *S. typhimurium* infection (Figures 1F,H) These results indicate that group 1 ILCs and NCR^+^ILC3s may play critical roles in immune responses against intracellular bacterial infections and suggest that the transcription factor Runx3 may participate in these processes.

### Runx3 participates in ILC1-mediated control of hepatic *L. monocytogenes* infection

To investigate the role of Runx3 in mediating group 1 ILCs and NCR^+^ILC3s against intracellular bacterial infection, we generated PLZF-cre Runx3 cKO mice (Figure S1A) (PLZF-cre Runx3 ^fl/fl^ are hereafter referred to as Runx3 cKO mice). It has been reported that PLZF is expressed in NKT cells during their development (27), we found the NKT cells number in thymus from PLZF-cre was decreased and those NKT cells exhibited more naïve phenotype (Figure S1B). In addition, the expression of PLZF was decreased in both stage1 and stage2 during NKT development (Figure S1C). In the Runx3 cKO strain, we observed efficiently deleted Runx3 in group 1 ILCs from the liver (Figure S1D), as well as in group1 ILCs and NCR^+^ ILC3s from the lamina propria of the small intestine (Figure S1F). The expression of Runx3 in the cKO group 1 ILCs from the liver was 73% less than the control (Figure S1E). Additionally, Runx3 in the cKO group 1 ILCs and NCR^+^ ILC3s dropped to 41% of the level in control littermates' small intestine lamina propria (Figure S1G).

We did not observe any alternations in common lymphoid precursors (CLP), common helper innate lymphoid cells (CHILP), immature ILC2s (iILC2) or NKps in the bone marrow of Runx3 cKO mice (Figure S2A). The number of cells in the early development lineage for ILCs in the bone marrow, including CLP, CHILP, iILC2, and NKp, were also comparable to the Runx3 cKO and control mice, which suggests that Runx1 and other proteins of the Runx family may have compensatory roles in the early development of ILCs in the bone marrow.

**Figure 2:**
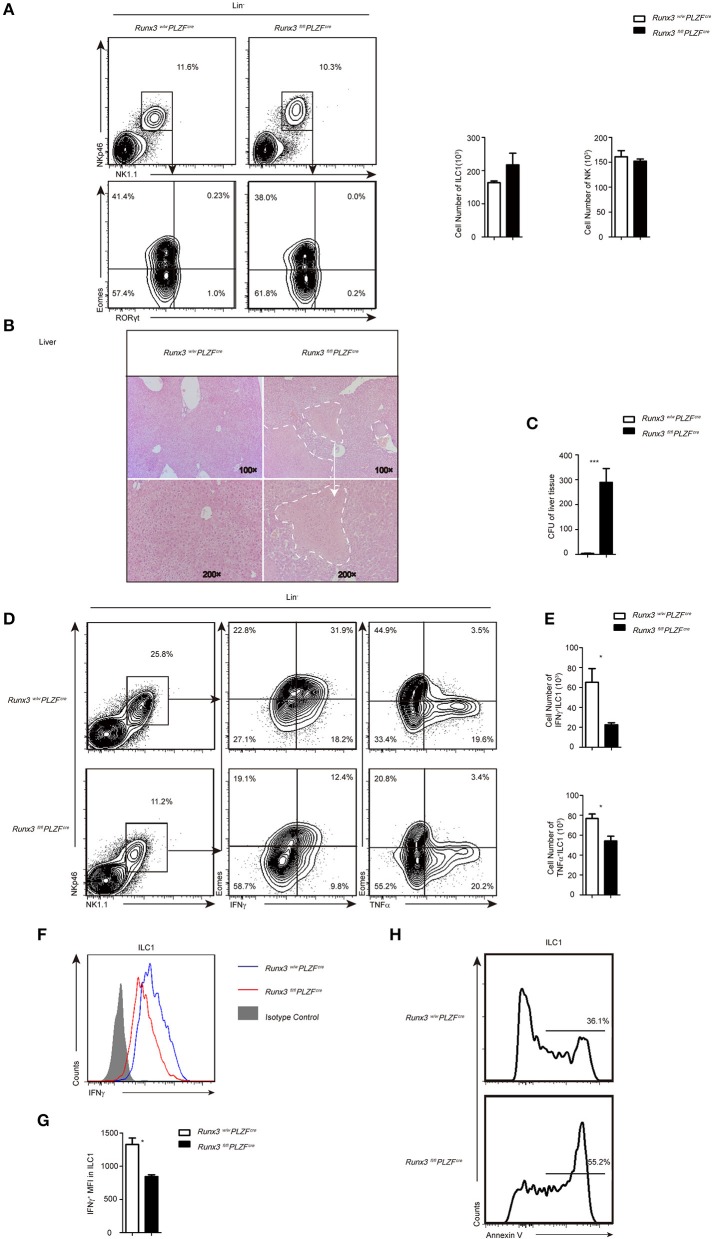
Increased sensitivity to *L. monocytogenes* infection partially due to defective ILC1 function after Runx3 deletion. **(A)** Flow cytometry analysis of the percentage of ILC1s and NK cells in the liver from control and cKO mice before infection, and absolute cell number of the indicated ILC population. **(B–H)** Control and cKO mice were infected with *L. monocytogenes* through tail vein injection (*n* = 6 per group). **(B)** Hepatic histology of livers obtained from wild and cKO mice stained with haematoxylin and eosin. **(C)** Titres of *L. monocytogenes* in the liver were measured 48 h after infection. **(D–G)** Cells isolated from the livers of infected wild type or cKO mice were stimulated with PMA/ionomycin and BFA for 4 h. **(D)** Flow cytometry assay of intracellular IFNγ from ILC1s in the middle and intracellular TNFα on the right. **(E)** Absolute cell number of the indicated cell types from wild type or cKO mice in the liver. **(F)** The intracellular expression of IFNγ in ILC1s. Isotype controls are shaded curves, wild type groups are blue curves and cKO groups are red curves. **(G)** Mean fluorescence intensity (MFI) of IFNγ in ILC1s. **(H)** Apoptosis of liver ILC1s labeled with annexin V (*n* = 3) (mean ± SD of three samples in b, d and f; ^*^P < 0.05; ^**^P < 0.01 by Student's *t*-test). Data are from one experiment representative of five independent experiments with similar results in **(A–F)**, and two independent experiments with similar results in **(G)**.

In contrast to the identical number of ILC1s and NKs in the liver (Figure 2C), both the number and percentage of NK1.1^+^ NKp46^+^ Lin^−^ cells were significantly reduced in the sLPL. In further studies, we found that RORgt^+^ NK1.1^+^ NKp46^+^ Lin^−^ (NK1.1^+^ NKp46^+^ ILC3) but not Eomes^−^ NK1.1^+^ NKp46^+^Lin^−^ (ILC1) or Eomes^+^NK1.1^+^ NKp46^+^ CD3^−^ CD19^−^ (NK) cells caused the observed reduction (Figure S2B). The expression of T-bet, a transcription factor that promotes the development of ILC1s and NKs, was unchanged (Figure S2E). Moreover, the number of RORγt^+^ ILC3 was also decreased, and this decrease was mainly due to a reduction of the total levels of NCR^+^ RORγt^+^ ILCs (NCR^+^ ILC3) (Figure S2C), and the expression of RORγt was also decreased (Figure S2D).

Runx3 is an important transcription factor that can moderate the development of T cells (28); therefore, we analyzed the phenotype of T cells in lymphoid nodes from both cKO and control mice and found a slight decrease in both the frequency and number of CD8^+^ T cells but not CD4^+^ T cells in the cKO mice (Figures S3A,B). In addition, as Runx3 was reported to play a role in triggering the secretion of IFNγ (29), we determined that the percentage cells expressing Runx3 in the Th1 cells was 59% and 15% in CD8^+^ T cells (Figures S3C,D). Furthermore, the secretion of IFNγ and TNFα by Th1 and CD8^+^ T cells was almost unchanged (Figures S3E,F).

At 48 h after *L. monocytogenes* infection, analysis of the liver histology showed severe hepatic necrosis in Runx3 cKO mice, whereas the livers of the control mice showed little damage (Figure 2A). Consistent with severe hepatic necrosis, the liver tissue of Runx3 cKO mice had much higher bacterial counts (Figure 2B). In contrast to the identical number of ILC1s and NKs in the liver before infection (Figure 2C), we observed that 48 h after infection, the number of ILC1s was decreased (Figure 2D) and ILC1s also showed impaired secretion of IFNγ (Figures 2E,F), a cytokine that mainly functions during infection by intracellular bacteria. The secretion of IFNγ decreased to approximately 63% of the wild littermates (Figure 2G). In addition, the ILC1s also showed decreased secretion of TNFα (Figure S4A), which is reported to have a protective role in intracellular bacterial infections. Moreover, ILC1s exhibited high levels of apoptosis (Figure 2H), which may explain the reduced number of cells in the Runx3 cKO mice after *L. monocytogenes* infection. In addition, Runx3 cKO mice showed impaired function of liver NKs. In the liver, we detected IFNγ and TNFα production defects (Figures S4A–C) and increased apoptosis (Figure S4D) after *L. monocytogenes* infection.

In addition, we determined the role of T cells and NKT cells in this *L. monocytogenes* infection model. We analyzed IFNγ production by CD4^+^, CD8^+^ T cell and NKT cells and found that the number of CD8^+^ T cells was decreased, whereas CD4^+^ T cells were unchanged in the livers from cKO mice (Figure S4E). The number of NKT cells was decreased a lot in cKO mice and the control mice, due to the disrupted zbtb16 allele in the PLZFcre mice, so we cannot rule out Runx3′ function in this cell population (30). Further studies showed that IFNγ production by CD4^+^, CD8^+^ T cell and NKT cells from cKO mice were all comparable to control mice (Figure S4F), although we cannot exclude the effect of the decreased number of CD8^+^ T cells.

### Runx3 promotes the function of intestinal group 1 ILCS and NCR^+^ILC3s against *S. typhimurium* infection

**Figure 3:**
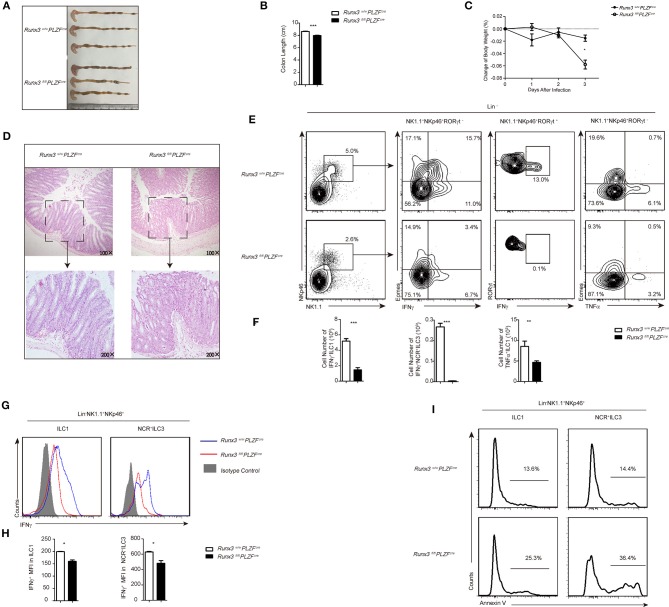
Defective function of ILC1s and NCR^+^ILC3s resulted in sensitivity to *S. typhimurium* infection after Runx3 deletion. **(A–I)** Control and cKO mice were orally infected with *S. typhimurium* (*n* = 6 per group). **(A,B)** Colon length, **(C)** change of bodyweight and **(D)** colon histology stained with haematoxylin and eosin were measured 4 days after oral infection before sacrifice. **(E–H)** LPL cells isolated from infected wild type or Runx3 KO mice were stimulated and a flow cytometry assay was performed 4 h later. **(E)** Intracellular IFNγ and TNFα in intestinal ILC1s was are the second and fourth line, respectively, and intracellular IFNγ in intestinal NCR^+^ILC3s is the third line. **(F)** Absolute cell number of the indicated cell types from wild type or cKO mice in the intestines after infection. **(G)** A flow cytometry assay was performed and the intracellular IFNγ produced by intestinal ILC1s and NCR^+^ILC3s was measured. Isotype controls are shaded curves, control groups are blue curves and cKO groups are red curves. **(H)** Mean fluorescence intensity (MFI) of IFNγ in the indicated cell types. **(I)** Apoptosis of intestinal ILC1s and NCR^+^ILC3s labeled with annexin V. (mean ± SD of three samples in **(B–D,F,H,I)**; ^*^P < 0.05, ^**^P < 0.01 and ^***^P < 0.001 by Student's *t*-test). Data are from one experiment representative of five independent experiments with similar results in **(A–H)**.

To further address the function of Runx3 in intestinal group 1 ILCs and NCR^+^ILC3s during intracellular bacterial infection, we used the *S. Typhimurium* infection model. Compared to control mice, Runx3 cKO mice were unable to prevent the enhanced bacterial translocation characterized typified by shortening of the colon (Figure 3A), which showed significant differences between the two groups (Figure 3B). At the same time, we observed more severe weight loss (Figure 3C) in the Runx3 cKO mice, indicating that in the absence of Runx3, mice could prevent enhanced bacterial translocation. In addition, histological analysis of Runx3 cKO mice showed more severe colon damage, including clearer epithelial injury, crypt hyperplasia and increased infiltration of inflammatory cells (Figure 3D).

We determined that in the absence of Runx3, ILC1s exhibited reduced production of IFNγ, suggesting that Runx3 participates in promoting antibacterial cytokine production. Furthermore, NCR^+^ ILC3s also exhibited reduced IFNγ secretion in Runx3 cKO mice, thereby acting as another source of IFNγ in small intestine ILC populations (Figures 3E,F). Moreover, the mean fluorescence intensity of IFNγ in ILC1s and NCR^+^ ILC3s was also lower after deletion (Figures 3G,H), and TNFα-producing ILC1s were reduced (Figures 3E,F and Figure S5A). We also observed increased apoptosis of ILC1s and NCR^+^ ILC3 after infection, which may explain the reduced number of ILC1s after infection (Figure 3I). Moreover, Runx3 cKO mice showed impaired function in intestinal NKs. In the small intestine, NK cells showed decreased IFNγ secretion (Figures S5B,C) and increased apoptosis (Figure S5D) after *S. typhimurium* infection.

Collectively, these data indicate that Runx3 participates in the role of ILC1s and NCR^+^ILC3s to control intestinal *S. typhimurium* infection by promoting the secretion of IFNγ. The deficiency of Runx3 likely accelerates the apoptosis of ILC1s, subsequently sensitizing the intestine to *S. typhimurium* infection.

### Runx3 promotes the function of intestinal group 1 ILCs and NCR^+^ILC3s against *S. typhimurium* infection in a cell intrinsic manner

It was reported that various types of immunocytes, namely, T cells, B cells, NK cells, and monocytes, may be involved in protection of the gut from pathogens. To further confirm that the impaired function of ILCs upon *S. typhimurium* infection was a direct result of the deletion of Runx3, we sorted 6000 NK1.1^+^NKp46^+^ILC cells from both control and Runx3 cKO mice. These cells were stimulated in the presence of IL7, IL12, and IL18 for 1 h before injecting into NCG mice. Due to the extremely low number of ILC1s in the gut, we performed adoptive transfer of sorted-ILC1 cells after stimulation on days −1, 0, 1, and 5 after *S. typhimurium* infection (31–33).

**Figure 4:**
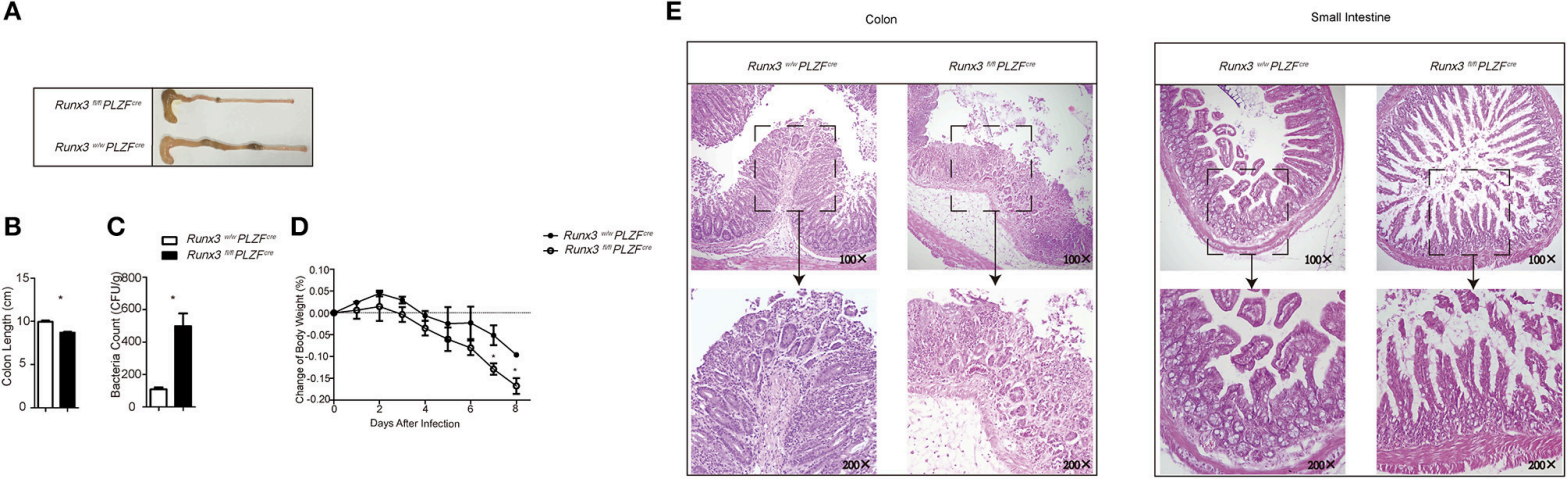
Cell intrinsic mechanism by which Runx3 promotes intestinal group 1 ILC and NCR^+^ILC3 function against *S. typhimurium* infection. **(A–E)** Intestinal ILC1s from control mice and Runx3 KO mice were isolated and stimulated with IL12 and IL18 at 40 ng/mL for 1 hour before injection into NCG mice through the tail vein at days −1, 0, 1, and 5 after lavage with *S. typhimurium*. **(A)** The length of the colon, **(B)** statistical analysis of the colon length, **(C)** titre of *S. typhimurium* in the deiecta, **(D)** change of body weight, and **(E)** histology of the small intestine (left) and colon (right) were determined 9 days after infection. (mean ± SD of three samples in k and m; ^*^P < 0.05 by Student's *t*-test). Data are from one experiment representative of two independent experiments with similar results in **(A–E)**.

Ten days after infection, the NCG mice injected with ILC1 cells sorted from Runx3 cKO mice exhibited significantly shorter colon length (Figures 4A,B). We also collected faces at day 5 post-infection and higher *S. typhimurium* colony counts were obtained from the faces of NCG mice injected with ILC1s sorted from Runx3 cKO mice (Figure 4C). We also observed faster loss of body weight in Runx3 cKO recipient mice (Figure 4D) and more severe damage to the gut wall (Figure 4E). Collectively, these data indicate that the increased sensitivity to *S. typhimurium* infection in Runx3 cKO mice occurs in a cell intrinsic manner.

### Runx3 depletion impairs the IL12-IL12R-pSTAT4 axis and affects the secretion of IFNγ in ILC1 and NCR^+^ILC3 cells

**Figure 5:**
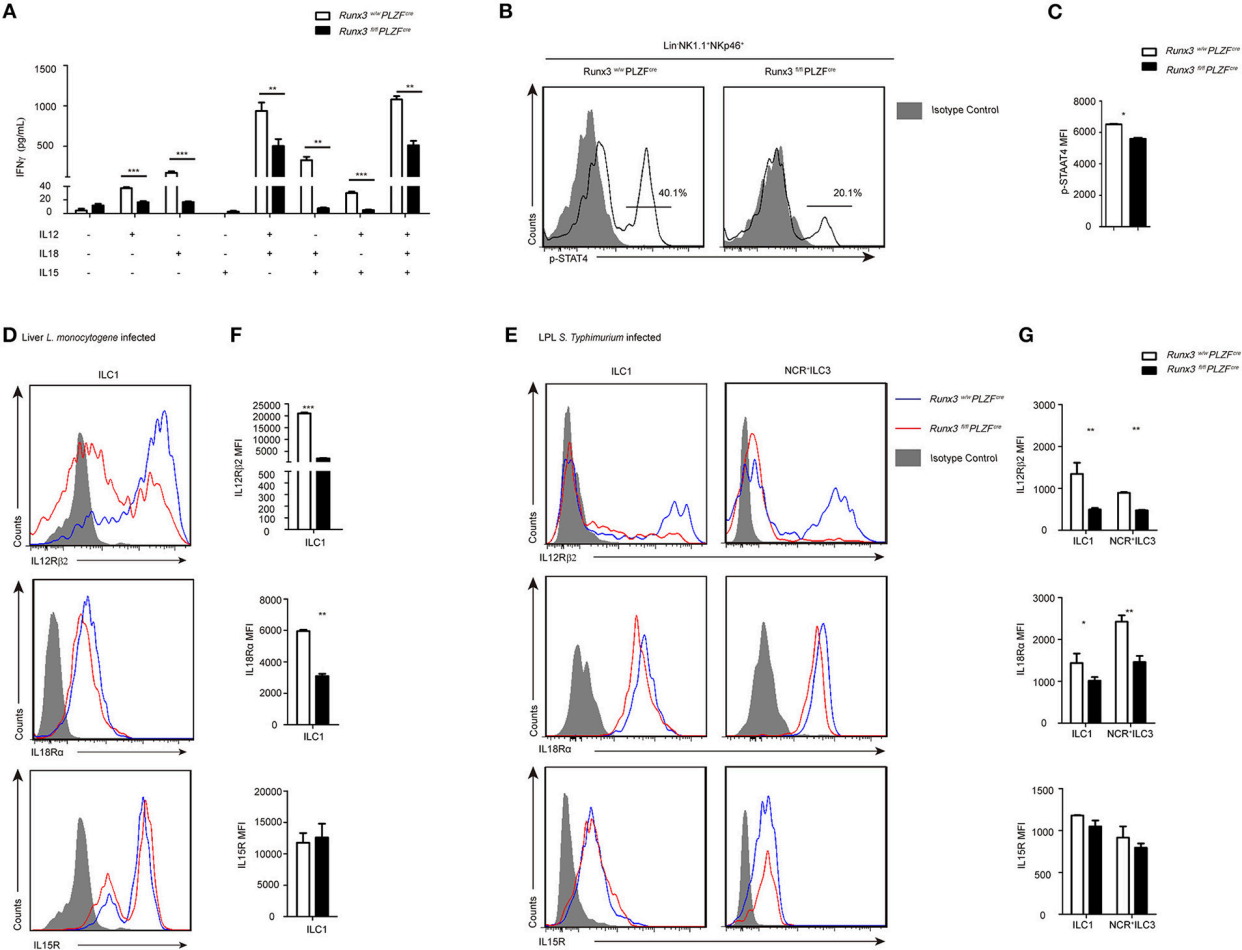
The IL12R signaling pathway was defective after Runx3 deletion in ILC1s and NCR+ILC3s. **(A)** ELISA for IFNγ secretion by intestinal ILC1s from wild type and Runx3 KO mice after stimulation with the indicated cytokines labeled under each bar for 36 h. **(B)** Flow cytometry assay of p-STAT4 in intestinal ILC1s from wild type and Runx3 KO mice after stimulation with IL12. **(C)** Statistical analysis of the change of p-STAT4. **(D,F)** Wild type and Runx3 KO mice were infected with *L. monocytogenes* through tail vein injection (*n* = 3 per group). **(D)** The expression of IL12Rβ2, IL18Rα and IL15R in ILC1s from the liver after *L. monocytogenes* infection and **(F)** mean fluorescence intensity (MFI) of the indicated proteins in ILC1s. **(E,G)** Control and Runx3 KO mice were orally infected with *S. typhimurium* (*n* = 6 per group). **(E)** The expression of IL12Rβ2, IL18Rα, and IL15R on ILC1s and NCR^+^ILC3s from the small intestine after *S. typhimurium* infection and **(G)** mean fluorescence intensity (MFI) of the indicated proteins in ILC1s and NCR^+^ILC3s (mean ± SD of three samples in **(A,C,F,G)**; ^*^P < 0.05, ^**^P < 0.01 and ^***^P < 0.001 by Student's *t*-test). Data are from one experiment representative of three independent experiments with similar results in **(A–C)** and four independent experiments with similar results in **(D–G)**.

IL12 and IL18 are interleukins that are naturally produced by dendritic cells, macrophages, and neutrophils in response to antigenic stimulation (7, 34, 35). They are strong stimulators of IFNγ in ILC1s. In addition, IL15 plays a role in promoting ILC1 survival and proliferation (36, 37). We sorted ILC1s from both wild control and Runx3 cKO mice and stimulated them with these cytokines. At 36 h after stimulation, the supernatant was collected to evaluate the concentration of IFNγ using ELISA. ILC1s with deleted Runx3 showed obvious decreases in IFNγ secretion when stimulated with IL12, IL18 or IL12 plus IL18. In addition, IL15 did not affect the production of IFNγ (Figure 5A). On the one hand, these results reveal that it is cell intrinsic, as opposed to environmental reasons, that lead to the defective secretion of IFNγ by ILC1s. On the other hand, it indicates that IL12R signaling may be deficient upon the deletion of Runx3 in ILC1s.

As previously reported, after the of binding IL12 to IL12R, STAT4 proteins are phosphorylated and accumulate in the promoter areas of *Ifng* to promote the expression of IFNγ (38). To explore the role of the IL12-IL12R-pSTAT4 axis in ILC1s after Runx3 deletion, we stimulated the sorted ILC1 and NCR^+^ILC3 cell from Runx3 cKO mice with IL12 and collected these cells to analyse the level of pSTAT4 by flow cytometry 1 h later. We found that the level of pSTAT4 clearly dropped after Runx3 deletion (Figures 5B,C). This result supports our hypothesis that the deletion of Runx3 may weaken the IL12-IL12R-pSTAT4 axis, thus affecting the secretion of IFNγ in ILC1s and NCR^+^ILC3s.

IL12R is composed of two different subunits, IL12Rβ1 and IL12Rβ2, but only IL12Rβ2 has the intracellular elements that lead to the phosphorylation of STAT4 and the initiation of downstream signaling (39). We infected Runx3 cKO mice and their control compartments with *L. monocytogenes* via tail vein injection or with *S. typhimurium* orally to analyse IL12Rβ2, IL18Rα, and IL15R expression in ILC1s and NCR^+^ILC3s. We found that ILC1s or NCR^+^ILC3s from both the liver and small intestine both expressed measurable levels of IL12Rβ2, IL18Rα, and IL15R after intracellular bacterial infection. After infection, the expression of IL12Rβ2 was decreased in ILC1s from the liver, and in ILC1s and NCR^+^ILC3s from the small intestine in Runx3 cKO mice. At the same time, the expression of IL18R was slightly decreased in ILC1s from the liver, and in ILC1s and NCR^+^ILC3s from the small intestine in Runx3 cKO mice. The expression of IL15R was not altered in these cells (Figures 5C–F). Moreover, after ablation of Runx3, NK cells also downregulated the expression of IL12Rβ2 (Figures S6A,B) in the liver, and both IL12Rβ2 and IL18Rα in the small intestine (Figures S6C,D). Moreover, the expression of IL15R was altered in NK cells from both the liver and small intestine.

### Runx3 directly binds to the promoter and intron 8 of IL12Rβ2 to affect the expression of IL12R in group 1 ILCS and NCR^+^ILC3s

**Figure 6:**
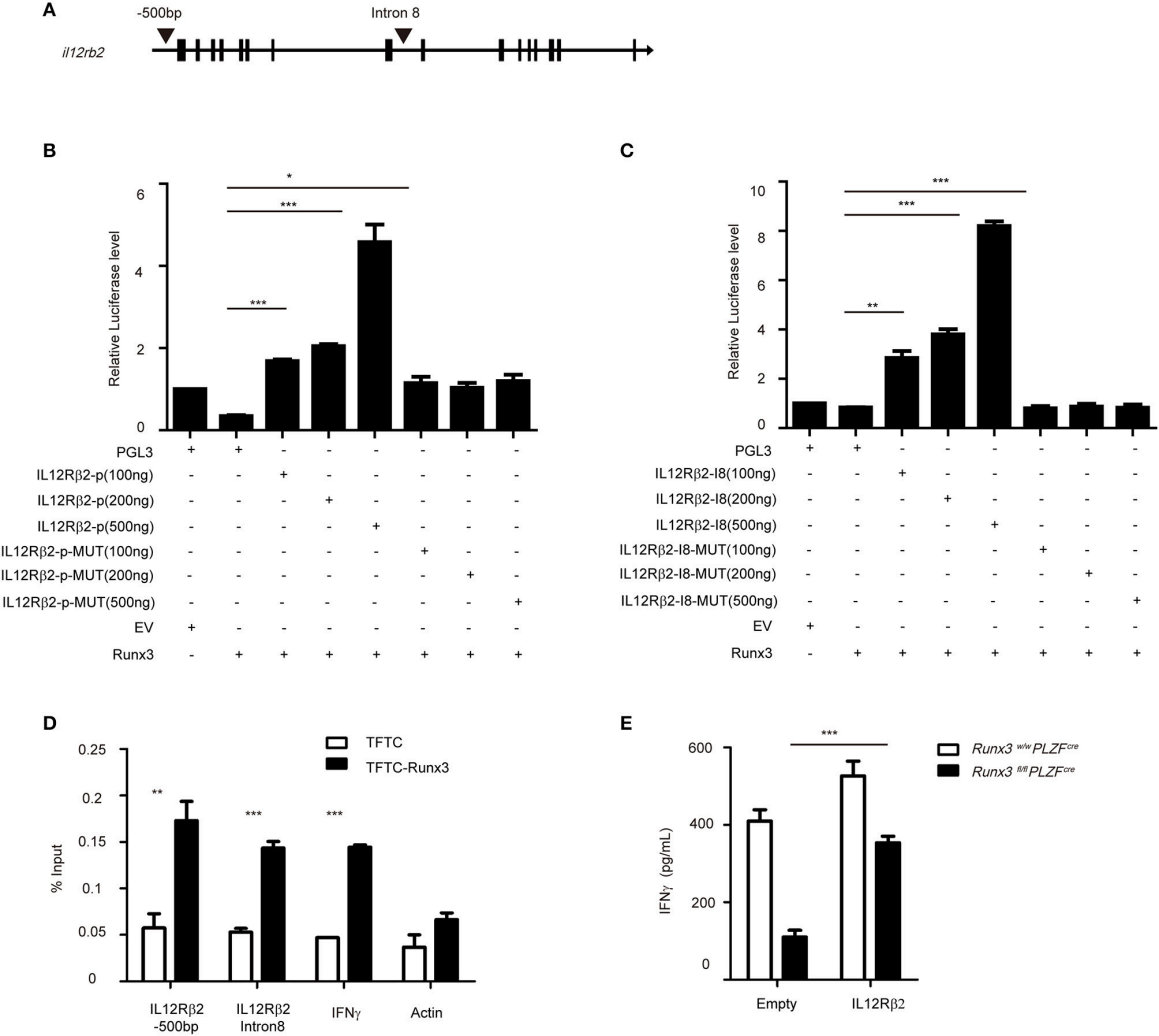
Runx3 directly binds to the promoter and intron 8 of *il12rb2* **(A)** Graph of the *il12r2* gene and location of Runx3 binding sites. **(B)** Luciferase activity in HEK293 cells transfected with various combinations (below plot) of vector alone (pGL3) or with 100, 200, or 500 ng of vector containing the wild-type promoter of *il12rb2 (il12rb2-p*) or with mutations in the Runx3-binding site of the promoter (*il12rb2-p-MUT*), together with empty vector (EV) or vector expressing Runx3 (Runx3). The results are presented relative to cells transfected with pGL3 and empty vector (far left), set to one. **(C)** Luciferase activity in HEK293 cells transfected with various combinations (below plot) of vector alone (pGL3) or with 100, 200 ng, or 500 ng of vector containing the wild-type intron 8 of *il12rb2 (il12rb2-I8*) or with mutations in the Runx3-binding site of the promoter (*il12rb2-I8-MUT*), together with empty vector (EV) or vector expressing Runx3 (Runx3). The results are presented relative to cells transfected with pGL3 and empty vector (far left), set to one. **(D)** Runx3 binding at the *il12rb2* locus in EL4 stable cell lines expressing either a triple flag peptide (TFTC) or triple-flag-tagged Runx3 (TFTC-RORγt) was monitored using a flag ChIP assay. The fold enrichment of Runx3 binding at each locus was normalized to TFTC-empty EL4 cells. The actin and *ifng* loci were used as negative and positive controls, respectively. **(E)** ILC1s were sorted from wild type and cKO mice and infected with retrovirus containing the indicated vector. ELISA for IFNγ secretion with the indicated vector overexpressed in ILC1s after stimulation with IL12 and IL18 for 36 h (mean ± SD of three samples in **(B–E)**; ^*^P < 0.05, ^**^P < 0.01 and ^***^P < 0.001 by Student's *t* test). Data are from one experiment representative of three independent experiments with similar results in **(A,C,D)** and two independent experiments with similar results in **(E)**.

Several labs published the conserved Runx3 binding motif as CCACA (29, 36). We found one conserved binding site at the promoter and another one in the intron 8 of *Il12R*β*2*, which is 269 bp upstream of a conserved T-bet binding site (Figure 6A). Therefore, we cloned the promoter and intron 8 of *Il12R*β*2*, which both contain the conventional Runx3 binding site, and performed luciferase reporter assays. We observed that after the addition of 100 ng Runx3 expression vector, the luciferase activity was 2-fold higher in EL4 cells transfected with vector containing the control promoter or intron 8 of *Il12R*β*2* than cells transfected with the empty vector. The luciferase activity was also much higher when co-transfected with 200 or 500 ng of the Runx3 expression vectors. Moreover, deletion of the Runx3-binding motif in the promoter and intron 8 of *Il12R*β*2* abrogated the enhancement of luciferase activity (Figures 6B,C). These results revealed that Runx3 promotes the expression of *Il12R*β*2* in a manner dependent on Runx3-binding motifs.

To elucidate the mechanism by which Runx3 modulates the expression of IL12Rβ2, we performed chromatin immunoprecipitation (CHIP) assays in the EL4 cell line, which over-expressed the Runx3-flag plasmid, and detected the binding of Runx3 to the promoter and intron 8 of *Il12R*β*2* (Figure 6D). We next rescued the decreased secretion of IFNγ via overexpression of IL12Rβ2 in group 1 ILCs from Runx3 cKO mice. After overexpression, we further stimulated the cells with IL12 and IL18 to analyse the levels of IFNγ secretion. This showed IL12Rβ2 reconstituted cells had restored IFNγ secretion (Figure 6E). This result further indicates that Runx3 promotes the secretion of IFNγ through the direct modulation of IL12Rβ2.

## Discussion

Although a considerable number of studies investigated the role of Runx3 in T-cell lineage commitment and function, as well as in ILC development, little is known about its function in ILCs against intracellular bacterial infection. After conditional knockout of Runx3, we found that mice were more sensitive to intracellular bacterial infection, namely, *S. typhimurium* and *L. monocytogenes*. This sensitivity was partially due to the reduced secretion of IFNγ by ILCs1 and NCR^+^ILC3s. Moreover, we revealed that after Runx3 deletion, IL12-STAT4 signaling was impaired in ILC1s and NCR^+^ILC3s from both the small intestine and liver. To explore the underlying mechanism, we performed CHIP in the EL4 cell line and showed that Runx3 can directly bind to the promoter region and intron 8 of the *Il12rb2* gene to promote its expression. In brief, in this study, we found that the Runx3-IL12-STAT4 pathway can modulate the secretion of IFNγ by group 1 ILCs and NCR^+^ILC3s after intracellular bacterial infection.

We also showed that group 1 ILCs and NCR^+^ILC3s accumulate in organs infected with intracellular bacteria and upregulate the expression of Runx3. We propose that it is through increased levels of proinflammatory cytokines, such as IL12 and IL18, in the infected organs that upregulate the expression of Runx3 in group 1 ILCs and NCR^+^ILC3s. Subsequently, the overexpression of Runx3 can then promote IL12-STAT4-IFNγ signaling to limit intracellular bacterial infection. A recent report showed that the promoter regions of *Runx1* and *Runx3* are targets of STAT4 and that STAT4 binding during NK cell activation induces epigenetic modifications of Runx3 gene loci, as STAT4 is a downstream protein of IL12 signaling (40). It is possible that this acts as a positive feedback loop via the Runx3-IL12-STAT4 pathway that promotes the secretion of IFNγ.

In T cells, there are several pathways that modulate the secretion of IFNγ, including the Runx3-dependent pathway (41, 42), the IL12-STAT4 pathway (43) and the T-bet-dependent pathway (44). There is evidence of cross talk between these three pathways. For example, T-bet can cooperate with Runx3 to bind to various elements of the *Ifng* gene (45), and high expression of T-bet can induce the increased expression of IL12Rβ2 to promote the IL12-STAT4 pathway (46), whereas the upregulation of T-bet may result in the increased expression level of Runx3 (45). In this report, our ELISA showed that after the deletion of Runx3, ILC1s had decreased secretion of IFNγ under the stimulation of IL12. In addition, we performed CHIP assays in the EL4 cell line and found direct binding of Runx3 at intron 8 of *Il12rb2*, a region that also contained a nearby T-bet binding site, in addition to identifying the direct binding of Runx3 to the *Ifng* promoter. These data reveal a novel IFNγ modulation pathway in ILC cells.

After the deletion of Runx3, the secretion levels of TNFα in ILCs from the liver and small intestine were decreased. We also found a smaller reduction of IL18Rα expression levels in group 1 ILCs and NCR^+^ILC3s compared with IL12Rβ2. It was reported that in T cells, IL12 signaling could promote the expression of IL18Rα (47). Therefore, we hypothesize that Runx3 defective ILCs may have weakened IL12 signaling and downregulated expression of IL18Rα. As a result, the downregulated IL18R may decrease the secretion of TNFα after infection via NF-κB signaling (47, 48).

Moreover, Runx3 cKO mice showed impaired function in both liver and intestinal NKs. We detected the IFNγ-production defect and increased apoptosis in the liver after *L. monocytogenes* infection. In the small intestine, NK cells also showed decreased IFNγ secretion and increased apoptosis after *S. typhimurium* infection. Upon ablation of Runx3, NK cells also downregulated the expression of IL12Rβ2 in the liver and IL12Rβ2 and IL18Rα in small intestine. These results indicate that Runx3 can also promote the function of NK cells in intracellular bacterial infections.

Unlike the phenotype of group 1 ILCs in Cbfβ ^f/f^ NKp46-cre mice, we observed no change in the number of group 1 ILCs in both the liver and small intestine. We propose that this may result from the compensation effect of Runx1 in the development of these ILCs. Furthermore, after infection, group 1 ILCs from both the liver and small intestine showed increased levels of apoptosis. It was reported that Runx3 can function as a nuclear regulator during interleukin-15-dependent activation of NK cells by regulating the expression of genes involved in cell survival and proliferation (36). We hypothesize that the deletion of Runx3 in group 1 ILCs impairs the expression of genes encoding anti-apoptotic factors downstream of IL15 to limit the level of apoptosis.

Zbtb16 encodes the transcription factor PLZF, which is expressed by ILCs during their development, and it is also expressed in Th1, CD8^+^ T cells and several invariant T cells, such as MAIT (49), Vg1^+^ Vd6.3^+^ gamma delta T cells (50) and iNKTs (27, 51). Therefore, the expression of Runx3 may be deficient in all cell types mentioned above from cKO mice. We cultured Th1 and CD8^+^ T cells and evaluated their IFNγ production (Figure S3). We concluded that in cKO mice, the deletion of Runx3 in Th1 and CD8^+^ T cells caused mild impairment to the production of IFNγ. Because T-bet and Runx3 are both expressed and cooperate to promote the secretion of IFNγ in Th1 (23) and CD8^+^ T cells (45), it is possible that T-bet accelerates IFNγ production in cKO mice. For iNKT cells, it was reported that the deletion of Runx3 did not affect the secretion of IFNγ in the liver (52). For the liver infection model, we compared IFNγ production by CD4^+^ T cells, CD8^+^ T cells and NKT cells in both cKO and control mice. We observed that in this infection model, IFNγ produced in both the cKO and control mice by CD4^+^ T cells, CD8^+^ T cells and NKT cells were not altered (Figures S4E,F). *In vitro* experiments demonstrated that Runx3 promotes IL12 signaling in Group 1 ILCs and NCR^+^ILC3s. Taken together, although we cannot rule out the role of several IFNγ producing invariant T cells in the liver infection model, the deletion of Runx3 in some PLZF expression cells other than ILCs is consistent with the conclusion that Runx3 mediates resistance to intracellular bacterial infection by promoting IL12 signaling in Group1 ILCs and NCR^+^ILC3s.

## Ethics statement

This study was carried out in accordance with the recommendations of the Institutional Animal Care and Use Committee of the Zhejiang University Laboratory Animal Center. The protocol was approved by the Institutional Animal Care and Use Committee of the Zhejiang University Laboratory Animal Center.

## Author contributions

LW and SY designed the research. SY, JY, LZ, BH, XG, WL, and CL performed the experiments. SY, XL, and LW wrote the manuscript. LL, DW, and JW provided expertise and advice. LW supervised the project.

### Conflict of interest statement

The authors declare that the research was conducted in the absence of any commercial or financial relationships that could be construed as a potential conflict of interest.

## Supplementary material

Figure S1 Conditional targeting of the mouse Runx3. **(A)** Strategy of PLZF-cre knockin mice. **(B)** Flow cytometry analyzed of percentage and stages of NKT cells in thymus. **(C)** Flow cytometry analyzed of the intracellular expression of PLZF in the indicated stage of NKT cells. **(D–F)** Quantification of Runx3 protein expression in ILC1 and NK from liver **(D,E)**; and ILC1, NK and NCR^+^ILC3 from intestine **(F,G)** from Runx3-cKO and control mice (mean ± SD of three samples in **(D–G)**; ^*^*P* < 0.05; ^**^*P* < 0.01; by Student's *t*-test). Isotype control in shaded curves, wild type control groups in dotted curves and cKO groups in solid curves. Data are from one experiment representative of three independent experiments with similar results in **(E,F)**; and two independent experiments with similar results in **(B–G)**.

Figure S2 Phenotype of Runx3 conditional knockout mice. **(A)** Flow cytometry analyzed of percentage of CLP, CHILP, iILC2, and NKp cells in bone marrow. And absolute cell number of indicated population. **(B)** Flow cytometry assay of ILCs from intestinal lamina propria. And absolute cell numbers of the indicated ILC populations. **(C)** Flow cytometry assay of intestinal ILC3. Total ILC3 was stained as Lin^−^RORγt^+^; and NCR^+^ILC3 as Lin^−^ RORγt^+^NKp46^+^. **(D)** The expression of RORγt in intestinal ILC3s from Runx3-cKO (red curve) and control (blue curve) mice. **(E)** The expression of T-bet in intestinal LC1, NK, and NCR^+^ILC3 from Runx3-cKO (red curve) and control mice (blue curve) (mean ± SD of three samples in **(B–E)**; ^*^*P* < 0.05; ^**^*P* < 0.01; by Student's *t*-test). Data are from one experiment representative of three independent experiments with similar results in **(B–E)**.

Figure S3 Phenotype and function of T cells from Runx3 conditional knockout mice. **(A)** Flow cytometry analyzed of percentage of T cells in lymphoid nods (*n* = 3). **(B)** Absolute cell number of indicated population. **(C)** The Quantification of Runx3 protein expression in Th1 and CD8^+^ T cells from Runx3-cKO (red curve) and control (blue curve) mice (*n* = 3). **(D)** The MFI of Runx3 in indicated cells. **(E)** Flow cytometry assay of intracellular IFNγ and TNFα in Th1 and CD8^+^ cells (*n* = 3). **(F)** Absolute cell number of indicated population (mean ± SD of three samples in **(B,D,F)**; ^*^*P* < 0.05; ^**^*P* < 0.01; by Student's *t*-test). Data are from one experiment representative of three independent experiments with similar results in **(A–F)**.

Figure S4 Defected function of liver NK, T cells and NKT cells after Runx3 conditional deletion. **(A–F)** Wild type control mice and Runx3 cKO mice were infected with *L. monocytogene* through the tail vein injection (*n* = 6 per group). Cells isolated from liver of infected wild type control or Runx3 cKO mice were stimulated with PMA/ionomycin and BFA for 4 h. **(A)** Flow cytometry assay of intracellular TNFα in ILC1s and NK; **(B)** Intracellular assay of TNFα (left) or IFNγ (right) in liver NK cells from wild type control or Runx3 cKO mice as in Figure 2C. **(C)** The expression of IFNγ in liver NK cell from Runx3 cKO (red curve) and control (blue curve) mice. **(D)** Apoptosis of liver NK labeled by annexin V (*n* = 3). **(E)** Flow cytometry assay of cell number of CD4^+^, CD8^+^ T cells, and NKT cells (*n* = 3). **(F)** Intracellular assay of IFNγ in liver CD4^+^, CD8^+^ T cells, and NKT cells from wild type control or Runx3 cKO mice (*n* = 3) (mean ± SD of three samples in **(B,D,E,F)**; ^*^*P* < 0.05; ^**^*P* < 0.01; by Student's *t*-test). Data are from one experiment representative of five independent experiments with similar result in **(A–C)**; two independent experiments with similar results in **(D)**.

Figure S5 Defected function of intestinal NK after Runx3 conditional deletion. **(A–D)** Wild type control mice and Runx3 cKO mice were infected with *S. Typhimurium* orally (*n* = 6 per group). Cells isolated from intestines of infected wild type control or Runx3 cKO mice were stimulated with PMA/ionomycin and BFA for 4 h. Flow cytometry assay of intracellular TNFα in ILC1s and NK **(A)**; **(B)** Intracellular assay of IFNγ in intestinal NK cells from wild type control or Runx3 cKO mice as in Figure 3E. **(C)** Intracellular assay of IFNγ in NK from Runx3 cKO (red curve) and control (blue curve) mice. **(D)** Apoptosis of intestinal NK labeled by annexin V (*n* = 3) (mean ± SD of three samples in **(B,D,F)**; ^*^*P* < 0.05; ^**^*P* < 0.01; by Student's *t*-test). Data are from one experiment representative of five independent experiments with similar result in **(A–C)**; two independent experiments with similar results in **(D)**.

Figure S6 Defected IL12Rβ2, IL18Rα, and IL15R expression on both liver and intestinal NK after Runx3 deletion. **(A,B)** Wild type control mice and Runx3 cKO mice were infected with *L. monocytogene* through the tail vein injection (*n* = 6 per group). **(A)** The expression of IL12Rβ2, IL18Rα, and IL15R on the NK from liver after infection and **(B)** Mean fluorescence intensity (MFI) of indicated proteins on NK after infection. **(C,D)** Wild type control mice and Runx3 cKO mice were infected with *S. Typhimurium* orally (*n* = 6 per group). **(C)** The expression of IL12Rβ2, IL18Rα, and IL15R on the NK from intestine after infection and **(D)** Mean fluorescence intensity (MFI) of indicated proteins on NK after infection (mean ± SD of three samples in **(B,D,F)**; ^*^*P* < 0.05; ^**^*P* < 0.01; by Student's *t*-test). Data are from one experiment representative of four independent experiments with similar results in **(A,C)**.
