# Supplementary figures and images for: Runx3 Mediates Resistance to Intracellular Bacterial Infection by Promoting IL12 Signaling in Group 1 ILC and NCR+ILC3
Source: Front Immunol. 2018 Sep 12;9:2101. doi: 10.3389/fimmu.2018.02101 (PMC6144956; doi:10.3389/fimmu.2018.02101)

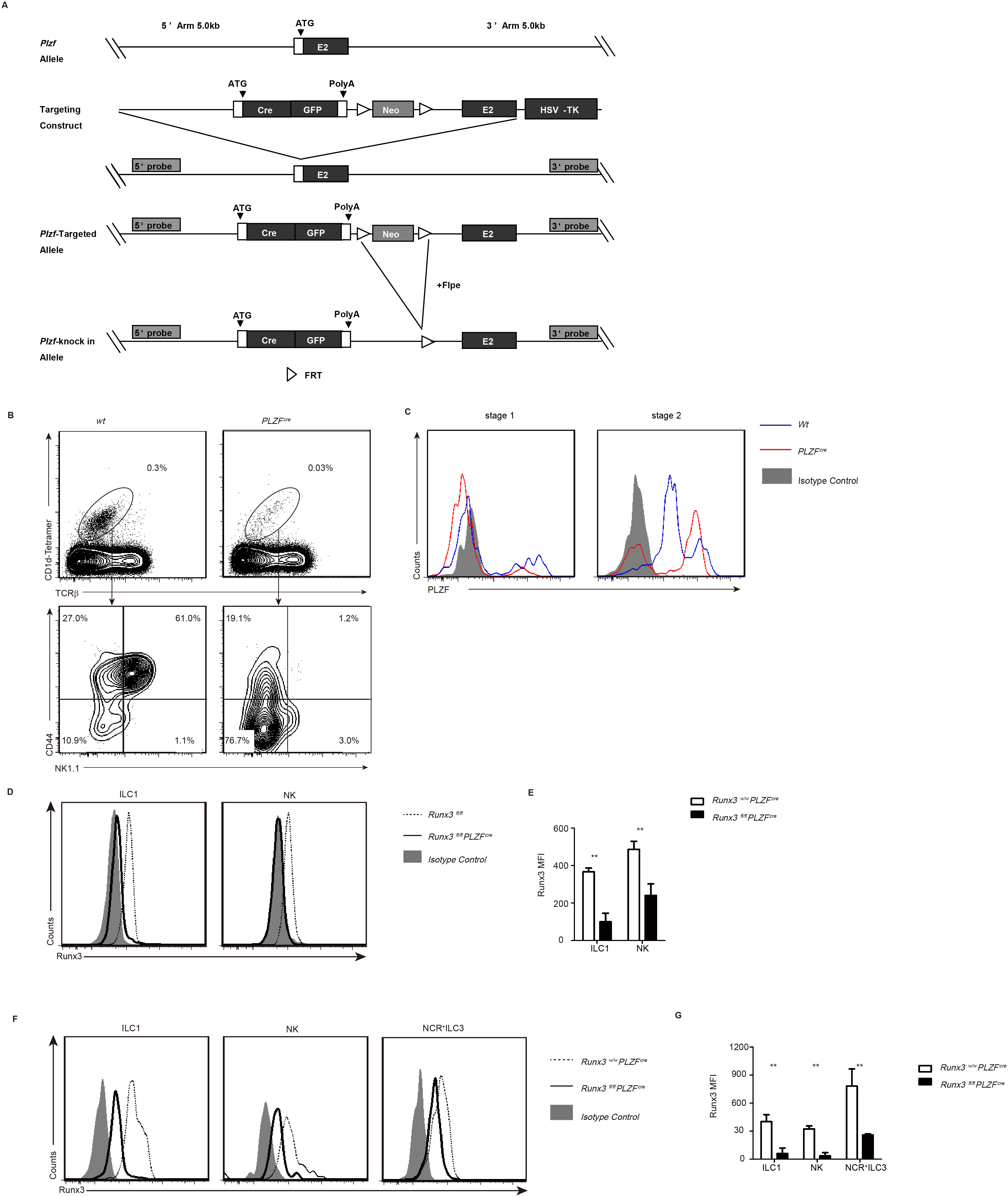

Supplement: Figure S1 — Conditional targeting of the mouse Runx3. (A) Strategy of PLZF-cre knockin mice. (B) Flow cytometry analyzed of percentage and stages of NKT cells in thymus. (C) Flow cytometry analyzed of the intracellular expression of PLZF in the indicated stage of NKT cells. (D–F) Quantification of Runx3 protein expression in ILC1 and NK from liver (D,E); and ILC1, NK and NCR+ILC3 from intestine (F,G) from Runx3-cKO and control mice (mean ± SD of three samples in (D–G); *P < 0.05; **P < 0.01; by Student's t-test). Isotype control in shaded curves, wild type control groups in dotted curves and cKO groups in solid curves. Data are from one experiment representative of three independent experiments with similar results in (E,F); and two independent experiments with similar results in (B–G). [file Image_1.TIF]

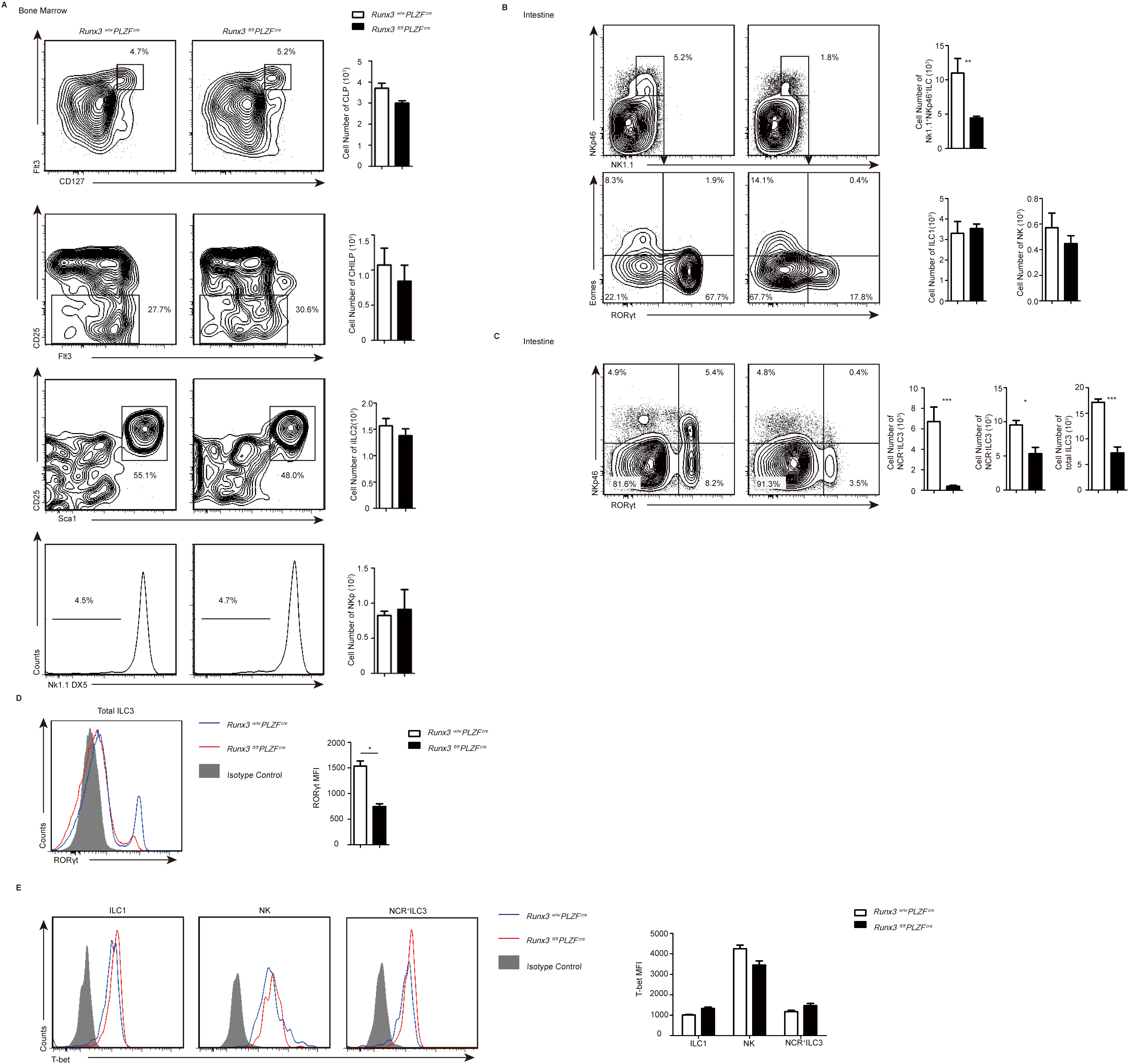

Supplement: Figure S2 — Phenotype of Runx3 conditional knockout mice. (A) Flow cytometry analyzed of percentage of CLP, CHILP, iILC2, and NKp cells in bone marrow. And absolute cell number of indicated population. (B) Flow cytometry assay of ILCs from intestinal lamina propria. And absolute cell numbers of the indicated ILC populations. (C) Flow cytometry assay of intestinal ILC3. Total ILC3 was stained as Lin−RORγt+; and NCR+ILC3 as Lin− RORγt+NKp46+. (D) The expression of RORγt in intestinal ILC3s from Runx3-cKO (red curve) and control (blue curve) mice. (E) The expression of T-bet in intestinal LC1, NK, and NCR+ILC3 from Runx3-cKO (red curve) and control mice (blue curve) (mean ± SD of three samples in (B–E); *P < 0.05; **P < 0.01; by Student's t-test). Data are from one experiment representative of three independent experiments with similar results in (B–E). [file Image_2.TIF]

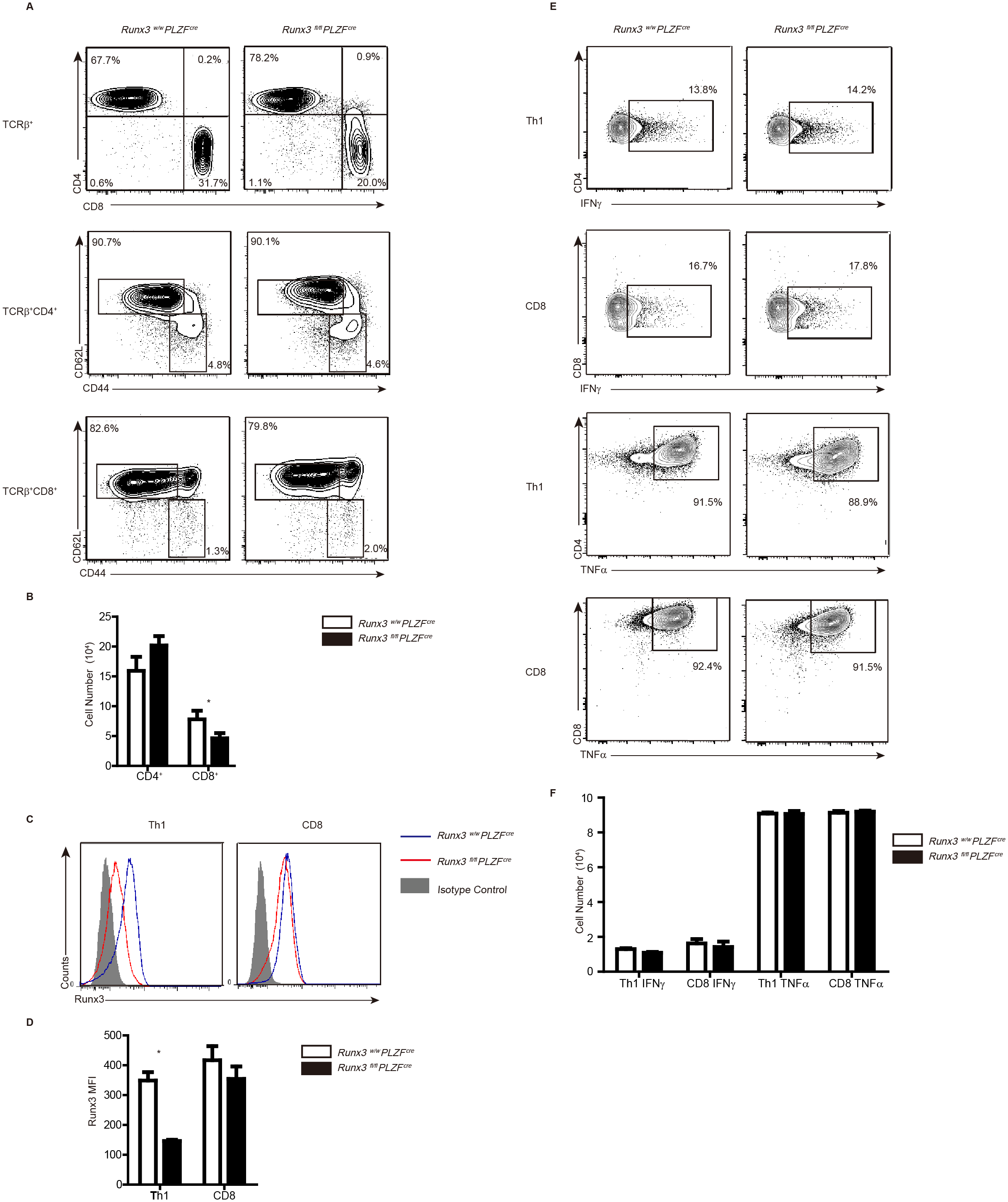

Supplement: Figure S3 — Phenotype and function of T cells from Runx3 conditional knockout mice. (A) Flow cytometry analyzed of percentage of T cells in lymphoid nods (n = 3). (B) Absolute cell number of indicated population. (C) The Quantification of Runx3 protein expression in Th1 and CD8+ T cells from Runx3-cKO (red curve) and control (blue curve) mice (n = 3). (D) The MFI of Runx3 in indicated cells. (E) Flow cytometry assay of intracellular IFNγ and TNFα in Th1 and CD8+ cells (n = 3). (F) Absolute cell number of indicated population (mean ± SD of three samples in (B,D,F); *P < 0.05; **P < 0.01; by Student's t-test). Data are from one experiment representative of three independent experiments with similar results in (A–F). [file Image_3.TIF]

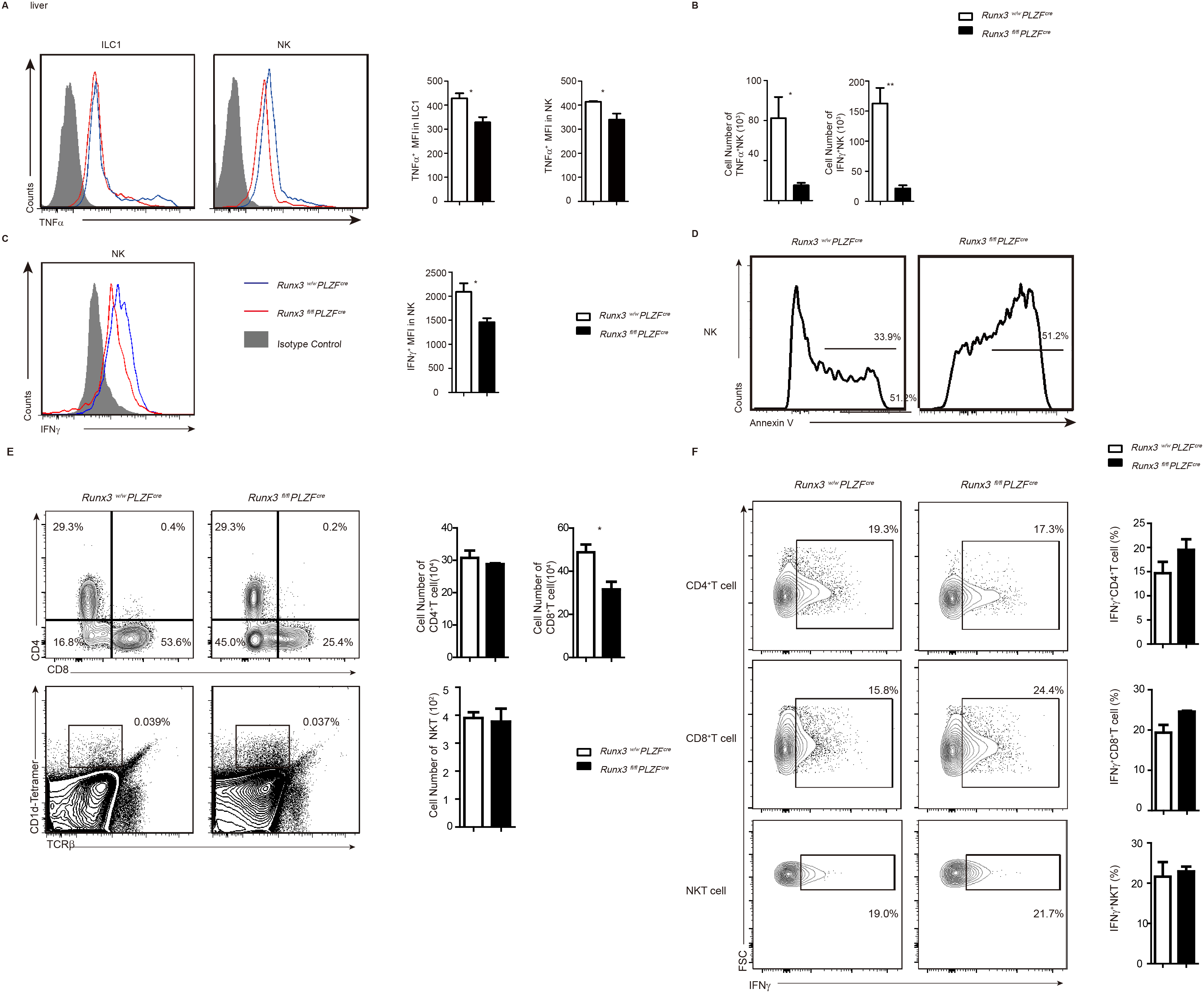

Supplement: Figure S4 — Defected function of liver NK, T cells and NKT cells after Runx3 conditional deletion. (A–F) Wild type control mice and Runx3 cKO mice were infected with L. monocytogene through the tail vein injection (n = 6 per group). Cells isolated from liver of infected wild type control or Runx3 cKO mice were stimulated with PMA/ionomycin and BFA for 4 h. (A) Flow cytometry assay of intracellular TNFα in ILC1s and NK; (B) Intracellular assay of TNFα (left) or IFNγ (right) in liver NK cells from wild type control or Runx3 cKO mice as in Figure 2C. (C) The expression of IFNγ in liver NK cell from Runx3 cKO (red curve) and control (blue curve) mice. (D) Apoptosis of liver NK labeled by annexin V (n = 3). (E) Flow cytometry assay of cell number of CD4+, CD8+ T cells, and NKT cells (n = 3). (F) Intracellular assay of IFNγ in liver CD4+, CD8+ T cells, and NKT cells from wild type control or Runx3 cKO mice (n = 3) (mean ± SD of three samples in (B,D,E,F); *P < 0.05; **P < 0.01; by Student's t-test). Data are from one experiment representative of five independent experiments with similar result in (A–C); two independent experiments with similar results in (D). [file Image_4.TIF]

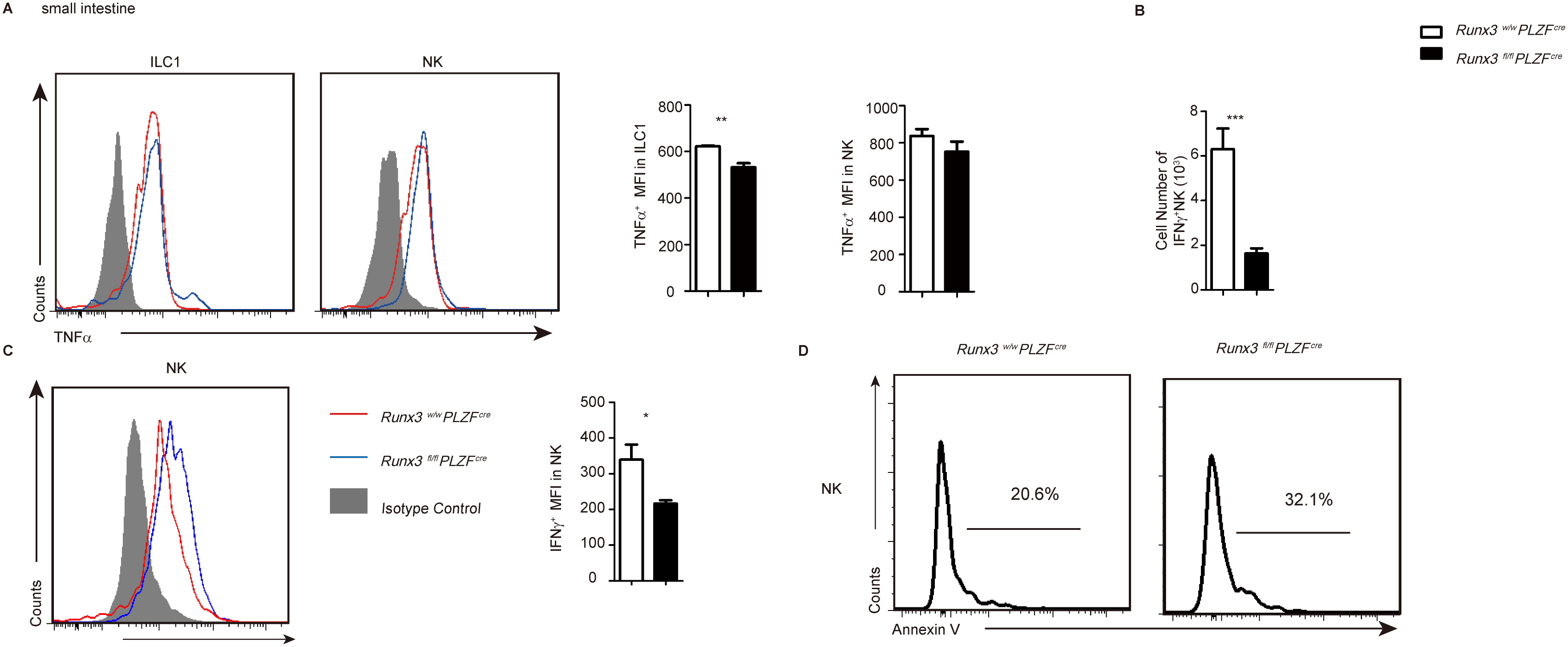

Supplement: Figure S5 — Defected function of intestinal NK after Runx3 conditional deletion. (A–D) Wild type control mice and Runx3 cKO mice were infected with S. Typhimurium orally (n = 6 per group). Cells isolated from intestines of infected wild type control or Runx3 cKO mice were stimulated with PMA/ionomycin and BFA for 4 h. Flow cytometry assay of intracellular TNFα in ILC1s and NK (A); (B) Intracellular assay of IFNγ in intestinal NK cells from wild type control or Runx3 cKO mice as in Figure 3E. (C) Intracellular assay of IFNγ in NK from Runx3 cKO (red curve) and control (blue curve) mice. (D) Apoptosis of intestinal NK labeled by annexin V (n = 3) (mean ± SD of three samples in (B,D,F); *P < 0.05; **P < 0.01; by Student's t-test). Data are from one experiment representative of five independent experiments with similar result in (A–C); two independent experiments with similar results in (D). [file Image_5.TIF]

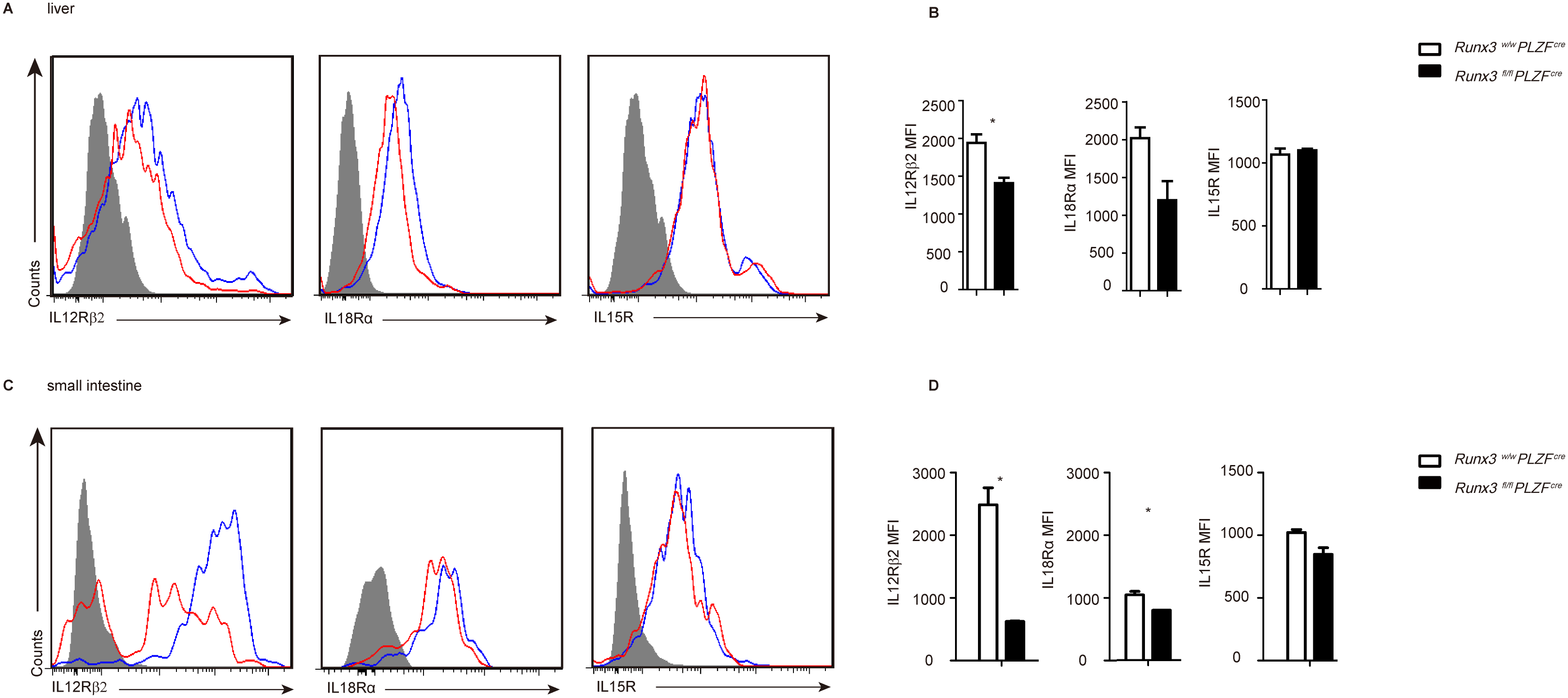

Supplement: Figure S6 — Defected IL12Rβ2, IL18Rα, and IL15R expression on both liver and intestinal NK after Runx3 deletion. (A,B) Wild type control mice and Runx3 cKO mice were infected with L. monocytogene through the tail vein injection (n = 6 per group). (A) The expression of IL12Rβ2, IL18Rα, and IL15R on the NK from liver after infection and (B) Mean fluorescence intensity (MFI) of indicated proteins on NK after infection. (C,D) Wild type control mice and Runx3 cKO mice were infected with S. Typhimurium orally (n = 6 per group). (C) The expression of IL12Rβ2, IL18Rα, and IL15R on the NK from intestine after infection and (D) Mean fluorescence intensity (MFI) of indicated proteins on NK after infection (mean ± SD of three samples in (B,D,F); *P < 0.05; **P < 0.01; by Student's t-test). Data are from one experiment representative of four independent experiments with similar results in (A,C). [file Image_6.TIF]
